# A systematic review and meta-analysis of the effects of inclusion of microalgae in dairy cows' diets on nutrient digestibility, fermentation parameters, blood metabolites, milk production, and fatty acid profiles

**DOI:** 10.5194/aab-69-101-2026

**Published:** 2026-02-10

**Authors:** Soumaya Boukrouh, Fadoua Karouach, Soufiane El Aayadi, Bouchra El Amiri, Jean-Luc Hornick, Abdelaziz Nilahyane, Abdelaziz Hirich

**Affiliations:** 1 African Sustainable Agriculture Research Institute (ASARI), Mohammed VI Polytechnic University (UM6P), Laâyoune 70000, Morocco; 2 Animal Production Department, Institut Agronomique et Veterinaire Hassan II, Medinat Al Irfane, Rabat, Morocco; 3 Department of Veterinary Management of Animal Resources, Faculty of Veterinary Medicine, University of Liège, 4000 Liège, Belgium

## Abstract

Recently, microalgae have been used as protein supplements to improve the productivity of dairy cows. However, the results are inconsistent among different studies. Thus, the aim of this study was to assess the effects of dietary microalgae incorporation on animal performance. The effect of microalgae was assessed by examining the raw mean differences (RMDs) between the treatment (with microalgae) and control (without microalgae) diets using a random-effect model. Heterogeneity was evaluated through meta-regression and subgroup analyses using microalgae species, inclusion level, days in milk, experimental duration, and cow breed as covariates. Microalgae supplementation decreased the intake of dry matter (DM), organic matter, and neutral detergent fiber (NDF). NDF digestibility improved, whereas the acetate : propionate ratio decreased. Milk and lactose yields remained unchanged. Despite a decrease in milk fat, the fatty acid (FA) profile improved, especially considering the increase in conjugated linoleic acid (CLA) C18:2 c9t11, docosahexaenoic acid (DHA) C22:6 n-3, and mono- and polyunsaturated FA (MUFA and PUFA) and the decrease in the n-6 : n-3 ratio. The main sources of variation in the responses to microalgal inclusion in cow milk production and quality were the animal breed, microalgae species, and their level of incorporation. In general, the incorporation of 61–100 g kg DM^−1^ of microalgae improved milk beneficial FA, including eicosapentaenoic acid (EPA) C20:5 n-3 and DHA, and *Schizochytrium* sp. increased DHA levels. The Holstein and Friesian breeds were characterized by a significant decrease in saturated FA (SFA). As a result, microalgae supplementation could be a sustainable agricultural practice for improving dairy cow milk quality.

## 1 Introduction

Ruminant milk is an important contributor to global food security, as it is a primary source of essential nutrients, including fat, protein, minerals, and vitamins (Moore et al., 2023). More precisely, dairy cows produce larger quantities of milk per individual than other ruminants, and their milk is widely consumed (Gross, 2022). As the world's population is predicted to rise, livestock farming systems are expected to encounter several obstacles, including feed scarcity, increased costs, rangeland degradation, emerging infectious diseases, and increased demand for animal products (Ait El Alia et al., 2025a, b; Boukrouh et al., 2025b, c, 2024b; Maass et al., 2012; Oosting et al., 2014). These challenges include the optimization of milk composition and feed efficiency while maintaining environmental sustainability. To improve milking performance, dairy cows require high amounts of concentrate and fodder as energy and protein sources, respectively. However, this solution intensifies competition and disputes over limited resources and accentuates the adverse consequences of climate change by polluting natural resources (Sakadevan and Nguyen, 2017; Tricarico et al., 2020). In this context, there is a pressing demand for alternative feed resources that are both environmentally sustainable and cost-effective while maintaining high-quality standards (Boukrouh et al., 2021, 2023b, c, 2024a; Hirich et al., 2020). Indeed, research is undergoing a substantial shift toward eco-friendly and low-cost practices to meet the increasing demand for high-quality dairy products (Tricarico et al., 2020). The investigation of alternative feed supplements has gained interest as a potential approach for enhancing dairy ruminant performance (Boukrouh, 2025). Owing to their distinct nutritional composition, microalgae have emerged as nutritional supplements for improving dairy cow milk production and quality (Valente et al., 2021). Microalgae provide a diverse array of essential nutrients, including lipids, minerals, proteins, and vitamins (Dolganyuk et al., 2020). Certain microalgal species are major sources of polyunsaturated fatty acids (PUFAs), particularly n-3 FA, making them attractive nutritional and pharmaceutical supplements and aquaculture feeds (Ferreira de Oliveira and Bragotto, 2022). Microalgae are promising alternatives because of their high productivity and nutrient content. These sources do not need fertile soil and can yield substantially more protein and fat than traditional feed options, which decreases the pressure on global agricultural resources. Furthermore, microalgae may be able to mitigate the negative environmental effects of other industries by using waste streams as nutrients. Investigating microalgae as a potential sustainable feed source offers an opportunity to address the current feed production limitations and foster a more secure and environmentally conscious food system (Fawcett et al., 2022).

Microalgae have been incorporated into ruminant diets in various countries as an alternative feed resource. However, the outcomes reported across studies are often inconsistent, reflecting differences in experimental conditions. Meta-analysis provides a robust quantitative approach to synthesize data from multiple studies. By combining smaller datasets, it increases statistical power, allows for the assessment of effect sizes, explores sources of heterogeneity, and can reveal reliable evidence of an intervention not detected in individual studies (Nakagawa et al., 2023). This method also supports the development of informed research strategies and feeding practices for future applications.

This meta-analysis hypothesized that incorporating microalgae into dairy cow diets would enhance milk production and quality. Several studies have provided interesting results; however, the literature remains diverse, encompassing variations in microalgal species, incorporation levels, and methodologies. Therefore, this study aimed to analyze the current state of knowledge through meta-analysis and offer a more comprehensive perspective that not only enlightens the scientific community but also guides dairy farmers and industry actors to optimize the nutrition of dairy cows for sustainable and improved milk production.

## 2 Materials and methods

### 2.1 Datasets

**Figure 1:**
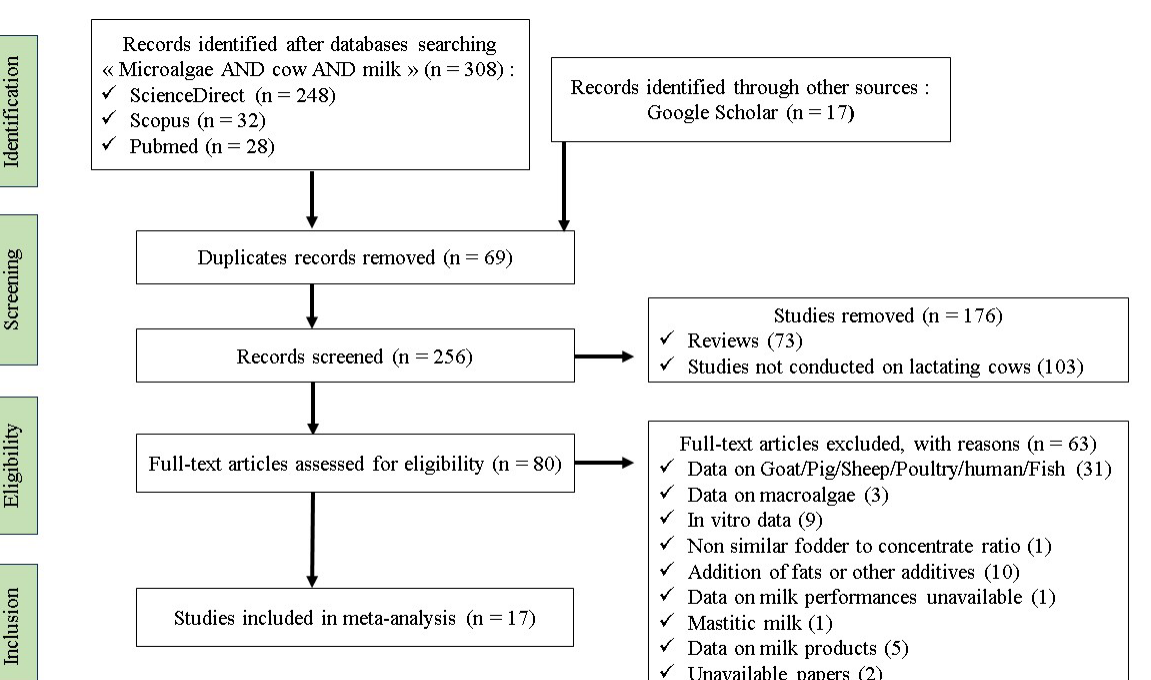
Flowchart of article selection based on PRISMA protocol.

A systematic search of the literature published in English was conducted using the Scopus, PubMed, ScienceDirect, and Google Scholar databases (Al Rharad et al., 2024). The identification, screening, eligibility assessment, and inclusion of studies adhered to the preferred reporting items for systematic reviews and meta-analyses (PRISMA) guidelines (Fig. 1) (O'Dea et al., 2021). This study was not restricted by date as it represents an inaugural attempt to compile data on the impact of microalgae on the lactation performance of dairy cows.

### 2.2 Datasets

Approximately 308 search papers were identified using “microalgae”, “cow”, and “milk” keywords, and duplicate publications were deleted using Zotero (https://www.zotero.org/, last access: 24 January 2024). Only studies meeting the predefined inclusion criteria were considered in this meta-analysis, namely (1) in vivo trials with healthy dairy cows during lactation, (2) studies reporting both experimental and control treatments that were fed microalgae, (3) diets that were free of oil and feed additives, and (4) data on the measure of variance. Some studies were excluded from the meta-analysis because either the fodder-to-concentrate ratio was not similar between the dietary groups or data were unavailable. A flowchart describing the process of screening studies for analysis is shown in Fig. 1. A total of 17 papers satisfied the inclusion criteria, and the details are presented in Supplement Table S1.

### 2.3 Data extraction

Based on the inclusion criteria, 17 studies were rated based on publication reference (first author and year), country or continent, experimental duration, days in milk, microalgae species, animal breed, and inclusion level in the diet. Only variables documented in a minimum of three separate publications were included in the meta-analysis (Sierra-Galicia et al., 2023). Data on the number of repetitions in the control and experimental groups and standard deviation (SD) were extracted. Duplicate data from papers published in more than one journal were excluded. When different incorporation levels were used in the same study, each was considered a treatment and compared to the same control.

### 2.4 Statistical analyses

Statistical analysis of the extracted data was performed using meta-analysis. The data were analyzed using the Metafor package of R software version 3.4.2 (Viechtbauer, 2010). The raw mean difference (RMD) with a 95 % confidence interval (CI) was used to calculate the effect of microalgal supplementation on dairy cattle performance and milk quality (Boukrouh et al., 2025a). The advantage of using the RMD is the expression of the effect size with a similar unit to the original measurement. The presence of heterogeneity or variation among studies was assessed using two statistical methods: the chi-square ( Q ) test and the $I^{2}$ statistic. Heterogeneity was considered to exist when the probability value fell below 0.05 (Deeks et al., 2019). When $I^{2}$ values fell below 25 %, minimal heterogeneity was indicated. A range of 25 % to 50 % suggests moderate heterogeneity, while values exceeding 50 % indicate high heterogeneity. Given that heterogeneity typically exists at varying degrees in each pooled analysis, we opted for a random-effect model for this study.

Egger's linear regression asymmetry test was employed to identify the presence of publication bias (Egger et al., 1997) for all outcomes and was considered significant when P≤0.05 . When this parameter confirmed the presence of publication bias, Rosenthal's Fail Safe Number (FSN) test was employed. FSN are generally considered robust when they are higher than 5 × Ne + 10, where Ne is the number of studies for each treatment group (Rosenthal, 1979). Subsequently, trim-and-fill analyses were conducted to determine whether the bias influenced the combined effect sizes and to detect the number of missing values (Duval and Tweedie, 2000). Funnel plots were used to represent asymmetry. In this case, publication bias exists if the distribution of the effect sizes around the true effect size is dissymmetric.

Meta-regression analysis was performed to determine the effects of categorical variables using a mixed model with the raw mean difference (RMD) as the dependent variable. The mixed models are defined as follows:

Θi=β+βixij+…+βiqxiq+μi,

where Θi is the true treatment effect in the i th explanatory variable, β is the whole true effect treatment, xij is the value of the j th variable ( j=1 , 2, …, q ) for the i th explanatory variable, βi is the difference in the true size effect for each unit increase in the j th variable, and μi∼N ( $0\;t^{2}$ ). The $t^{2}$ value represents heterogeneity not explained by the variable (Viechtbauer, 2010).

For the variable coefficients, null hypothesis tests were performed using the multiparameter Wald test (Harbord and Higgins, 2008). The adjusted $R^{2}$ was used to determine the extent to which the variables accounted for the variance between studies. This metric was computed by comparing the estimated between-study variance when the variables were included in the model ( $\sigma^{2}$ ) with the variance observed when these variables were omitted ( $\sigma o^{2}$ ).

$$\mathrm{Adjusted}\;R^{2}\;=\;((\sigma o^{2}-\sigma^{2}))/\sigma o^{2}\sigma o$$

The meta-regression approach criteria were as follows: (1) high heterogeneity ( $I^{2}$ statistic >50 %), (2) P value ≤ 0.05 for the heterogeneity test, and (3) P value ≥ 0.05 for Egger's test (Orzuna-Orzuna et al., 2021). The variables were categorized as follows: days in milk ( <90 or >90 d), experimental duration ( <100 , 100–200, >200 d), microalgae species (*Schizochytrium* sp., *Schizochytrium limancinum*, *Chlorella vulgaris, Japonochytrium* sp., *Spirulina platensis, Aurantiochytrium limacinum, Arthrospira platensis,* and *Prototheca moriformis*), microalgae inclusion levels ( <15 , 15–30, 31–60, 61–100, >100 g kg DM^−1^), and animal breed (Chinese–Friesian, Holstein, Friesian, Finnish, and Finnish Ayrshire). A covariate with a probability of less than 0.10 was considered to be the source of heterogeneity and was used in the subgroup analysis. A covariate for which the P value in the subgroup evaluation was less than 0.05 convincingly explained the observed heterogeneity.

## 3 Results

### 3.1 Characteristics of studies included in the meta-analysis

**Figure 2:**
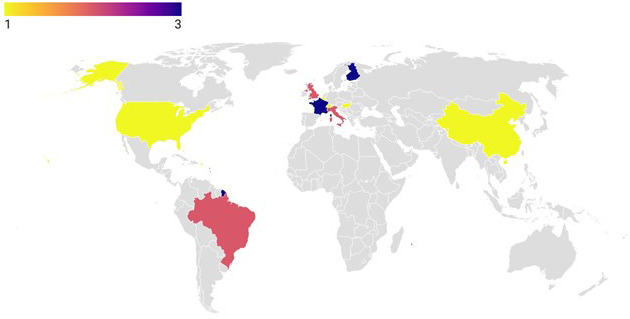
The geographic distribution of the included studies is illustrated on the map with generated latitude and longitude. Colored areas indicate the frequency of studies on a global scale.

**Figure 3:**
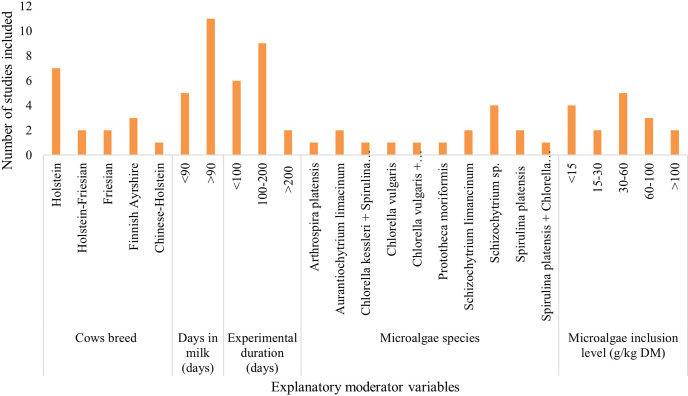
Bar chart illustrating the frequency of explanatory predictor variables employed in the meta-analysis.

The dataset included 17 peer-reviewed publications with 28 treatment means. The characteristics of the 17 studies included in this meta-analysis are detailed in Supplement Table S1. The data show that almost 90 % of the publications were published between 2015 and 2020. The geographical distribution of the studies by country in Fig. 2 indicates that the 17 studies spanned 10 countries, encompassing three continents (Asia, the Americas, and Europe). Supplement Table S1 and Fig. 2 show that the first attempt to incorporate microalgae into cow diets started in the USA in 1999, yet the focus on microalgae feed trials has recently been conducted mostly in Europe. A bar graph of the covariates considered shows that seven studies were conducted on the Holstein breed (Fig. 3). A total of 11 studies were conducted on cows with days in milk higher than 90 d. Nine experiments were handled over a medium-term period (100–200 d). Of the studies involved in the meta-analysis, four trials were conducted on *Schizochytrium* sp., and five trials used an inclusion level of 30–60 g kg DM^−1^.

### 3.2 Meta-analysis

This meta-analysis evaluated the effects of microalgal supplementation on dairy cow performance and milk quality (Tables 1 and 2). Microalgae supplementation significantly ( P<0.05 ) decreased the intake of dry matter (DMI; RMD = - 0.60 kg d^−1^; P=0.011 ), organic matter (OMI; RMD = - 0.43 kg d^−1^; P=0.024 ), and neutral detergent fiber (NDFI; RMD = - 0.26 kg d^−1^; P=0.002 ) but had no effect on the intake of protein (CPI) and ether extract (EEI). Microalgae improved only the digestibility of NDF (NDFD; RMD = 12.57 g kg DM^−1^; P=0.024 ) but had no effect on DM digestibility (DMD), OMD, and CPD. Microalgae inclusion in the diet improved isovalerate (RMD = 0.61 mol 100 mol^−1^; P=0.026 ) and decreased acetate : propionate (RMD = - 0.09; P=0.004 ) but had no significant effect on ruminal pH, acetate, valerate, isobutyrate, and ammonia. No significant effects were reported for blood parameters.

**Table 1:** Effects of microalgae supplementation in cow diets on feed intake, digestibility of nutrients, fermentation, and blood parameters.

Outcomes	Control mean (SD)	NC	Combined effect size		Heterogeneity		Egger
			Adjusted effect (95 % CI)	P value	P value	$I^{2}$ (%)	P value
Intake (kg d^−1^)							
Dry matter (DMI)	21.48 (6.12)	26	- 0.60 ( - 1.06, - 0.14)	0.011	<0.001	86.22	0.071
Organic matter (OMI)	12.25 (10.66)	14	- 0.43 ( - 0.80, - 0.06)	0.024	0.479	20.02	0.213
Crude protein (CPI)	3.78 (4.23)	15	- 0.05 ( - 0.18, 0.08)	0.464	0.002	66.93	0.887
Neutral detergent fiber (NDFI)	5.12 (4.52)	14	- 0.26 ( - 0.43, - 0.09)	0.002	0.027	55.02	0.047
Ether extract (EEI)	0.35 (0.72)	7	- 0.01 ( - 0.02, 0.01)	0.630	0.628	0	0.409
Digestibility parameters (g kg^−1^ DM)							
Dry matter (DMD)	679.54 (184.56)	13	2.09 ( - 4.70, 8.89)	0.546	<0.001	68.14	0.124
Organic matter (OMD)	693.00 (188.89)	13	2.85 ( - 4.17, 9.87)	0.427	<0.001	67.53	0.149
Crude protein (CPD)	657.54 (180.61)	13	3.58 ( - 5.04, 12.21)	0.416	<0.001	63.64	0.015
Neutral detergent fiber (NDFD)	526.31 (171.64)	13	12.57 (1.63, 23.50)	0.024	0.025	54.17	0.150
Fermentation parameters							
pH rumen	6.44 (2.46)	6	- 0.05 ( - 0.13, 0.04)	0.276	0.003	63.78	0.075
Acetate (mol 100 mol^−1^)	66.95 (25.52)	6	- 0.33 ( - 0.73, 0.08)	0.117	0.104	42.83	0.440
Isovalerate (mol 100 mol^−1^)	4.78 (3.34)	6	0.61 (0.07, 1.14)	0.026	<0.001	85.11	0.956
Valerate (mol 100 mol^−1^)	10.36 (6.10)	6	- 0.25 ( - 0.59, 0.09)	0.145	0.313	15.73	0.565
Isobutyrate (mol 100 mol^−1^)	7.08 (4.08)	6	0.09 ( - 0.26, 0.44)	0.614	0.064	51.37	0.386
Acetate : propionate	3.41 (1.56)	8	- 0.09 ( - 0.16, - 0.03)	0.004	0.028	55.22	0.834
Ammonia (NH_3_, mmol L^−1^)	5.52 (2.34)	6	0.50 ( - 0.43, 1.43)	0.290	0.026	61.17	0.324
Blood parameters							
Blood urea nitrogen (BUN; mg dL^−1^)	271.7 (120.6)	7	- 4.8 ( - 14.2, 4.6)	0.314	<0.001	85.49	0.992
Aspartate aminotransferase (AST; IU/L)	88.52 (40.15)	6	1.91 ( - 0.34, 4.16)	0.096	0.949	0	0.863
Glucose (mg dL^−1^)	60.58 (21.23)	18	- 0.23 ( - 1.16, 0.70)	0.626	<0.001	94.97	0.209

SD: standard deviation, NC: number of comparisons, RMD: raw mean difference, CI: confidence interval of RMD, P : probability, IU: international unit.

**Table 2:** Effects of microalgae supplementation to dairy cow diets on milk yield, chemical composition, and fatty acid profile.

	Control mean (SD)	NC	Combined effect size		Heterogeneity		Egger
			Adjusted effect (95 % CI)	P value	P value	$I^{2}$ (%)	P value
Yield (kg d^−1^)							
Milk	29.99 (7.83)	27	0.99 ( - 0.10, 2.07)	0.076	<0.001	92.73	0.452
Protein	0.99 (0.32)	26	- 0.02 ( - 0.04, 0.01)	0.199	<0.001	81.49	0.498
Lactose	1.20 (0.64)	20	- 0.004 ( - 0.03, 0.02)	0.781	0.017	20.85	0.505
Fat	1.20 (0.37)	25	- 0.07 ( - 0.12, - 0.02)	0.005	<0.001	84.82	0.352
Energy-corrected milk (ECM)	20.67 (16.39)	15	- 0.60 ( - 1.31, 0.13)	0.105	0.032	45.02	0.116
Composition (%)							
Protein	3.38 (0.70)	27	- 0.02 ( - 0.04, 0.004)	0.096	0.083	38.11	0.812
Lactose	4.69 (0.97)	25	- 0.05 ( - 0.07, - 0.03)	<0.001	0.580	0	0.679
Fat	4.08 (0.93)	27	- 0.16 ( - 0.28, - 0.04)	0.009	<0.001	74.75	0.592
Fatty acid profile (g 100 g FA^−1^)							
Lauric (C12:0)	3.57 (0.88)	19	- 17.07 ( - 50.27, 16.14)	0.314	<0.001	100	0.442
Myristic (C14:0)	11.67 (2.79)	19	0.05 ( - 0.25, 0.34)	0.766	<0.001	86.62	0.684
Pentadecanoic (C15:0)	1.25 (0.57)	15	7.56 ( - 7.62, 22.74)	0.329	<0.001	100	0.801
Palmitic (C16:0)	31.74 (9.14)	20	0.28 ( - 1.07, 1.62)	0.688	<0.001	96.72	0.245
Heptadecanoic (C17:0)	0.84 (0.87)	15	- 0.003 ( - 0.01, 0.00)	0.112	<0.001	0	<0.001
Stearic (C18:0)	9.72 (3.05)	20	- 1.09 ( - 2.24, 0.07)	0.066	<0.001	98.88	0.754
Myristoleic (C14:1)	1.24 (0.64)	13	0.03 ( - 0.02, 0.07)	0.211	0.034	12.78	0.005
Palmitoleic (C16:1)	1.76 (0.81)	13	- 0.15 ( - 0.30, - 0.004)	0.044	<0.001	67.29	0.452
Oleic (C18:1 n-9)	18.96 (4.99)	20	- 1.46 ( - 2.92, - 0.003)	0.050	<0.001	99.98	0.887
Linoleic (C18:2 n-6)	2.38 (0.73)	20	0.04 ( - 0.10, 0.17)	0.588	<0.001	100	0.043
Conjugated linoleic (CLA; C18:2 c9t11)	0.56 (0.34)	18	0.22 (0.10, 0.33)	<0.001	<0.001	97.02	0.427
α -linolenic (ALA; C18:3 n-3)	0.40 (0.21)	17	- 0.01 ( - 0.04, 0.01)	0.355	<0.001	98.08	0.002
Eicosapentaenoic (EPA; C20:5 n-3)	0.07 (0.04)	12	0.02 ( - 0.04, 0.07)	0.555	<0.001	99.77	0.234
Docosahexaenoic (DHA; C22:6 n-3)	0.03 (0.03)	14	0.30 (0.15, 0.45)	<0.001	<0.001	99.97	0.043
Total SFA	69.24 (15.87)	19	- 1.69 ( - 2.41, - 0.97)	<0.001	<0.001	85.31	0.151
Total MUFA	24.75 (6.08)	18	0.63 (0.26, 0.99)	<0.001	0.016	42.49	0.867
Total PUFA	3.52 (0.94)	19	0.50 (0.17, 0.83)	0.003	<0.001	98.13	0.101
n-3	0.58 (0.28)	17	0.12 (0.05, 0.19)	<0.001	<0.001	98.95	0.832
n-6	2.86 (1.36)	17	- 0.02 ( - 0.11, 0.06)	0.608	<0.001	85.87	0.389
n-6 : n-3	6.10 (4.49)	14	- 0.58 ( - 1.10, - 0.06)	0.028	<0.001	98.73	0.765

SD: standard deviation, NC: number of comparisons, RMD: raw mean difference, CI: confidence interval of RMD, P : probability, IU: international unit, MUFA: monounsaturated fatty acid, PUFA: polyunsaturated fatty acid, SFA: saturated fatty acid, n-3: omega-3 FA, n-6: omega-6 FA.

Microalgae supplementation had no significant effect on the yields of milk, protein, lactose, or the energy-corrected milk (ECM) but decreased fat yield (RMD = - 0.07 kg d^−1^; P=0.001 ), fat content (RMD = - 0.16 % DM; P=0.009 ), and lactose content (RMD = - 0.05 % DM; P<0.001 ). Microalgae incorporation into cow diets affected the milk fatty acid (FA) profile by significantly increasing conjugated linoleic acid (CLA) C18:2 c9t11 (RMD = 0.22 g 100 g FA^−1^; P<0.001 ) and docosahexaenoic acid (DHA) C22:6 n-3 (RMD = 0.30 g 100 g FA^−1^; P<0.001 ). Microalgae significantly improved FA summaries and ratios by increasing MUFA (RMD = 0.63 g 100 g FA^−1^; P<0.001 ), PUFA (RMD = 0.50 g 100 g FA^−1^; P=0.003 ), and n-3 (RMD = 0.12 g 100 g FA^−1^; P<0.001 ) and decreasing SFA (RMD = - 1.69 g 100 g FA^−1^; P<0.001 ) and n-6 : n-3 (RMD = - 0.58; P=0.028 ).

### 3.3 Meta-regression analysis and publication bias

**Figure 4:**
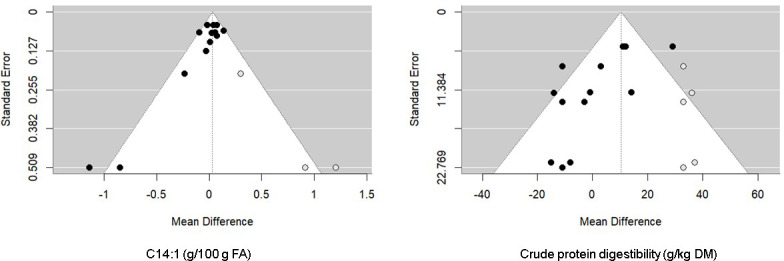
Funnel plot of the raw mean difference in studies (black circles) from all publications with C14:1 (left) and CPD (right). The empty circles represent the missing papers added after the trim-and-fill analysis.

As shown in Tables 1 and 2, heterogeneity was high for all parameters except for OMI, EEI, acetate, valerate, aspartate aminotransferase (AST), lactose yield and content, protein content, ECM, and total MUFA ( $I^{2}<50$ %). Publication bias was not evident for almost all parameters ( P>0.05 ). However, according to Egger's test, publication bias existed for NDFI, CPD, C18:1 t11, C18:2 n-6, C18:3 n-3, and C22:6 n-6 ( P<0.05 ). However, Rosenthal's FSN for the database were 281 (NDFI), 13 593 (C18:1 t11), 26 645 815 166 (C18:2 n-6), 66 (C18:3 n-3), and 26 660 (C22:6 n-3), which were higher than the Rosenthal FSN of 50 (5 × 8 + 10), 70 (5 × 12 + 10), 70 (5 × 12 + 10), 65 (5 × 11 + 10), and 65 (5 × 11 + 10) required to declare a significant mean effect size, despite the possibility of publication bias (Rosenthal, 1979). In contrast, Rosenthal's FSN 3 (CPD) and 0 (C14:1) were lower than the mean effect sizes of 45 (5 × 7 + 10) and 30 (5 × 4 + 10), respectively. For the latter variable, the trim-and-fill method revealed five missing observations for CPD and three missing observations for C14:1 in the funnel plot (Fig. 4).

To investigate the primary factors contributing to variability in the response variables, a meta-regression analysis was conducted. The results are presented in Table 3. Among the covariates, cow breed, microalgae species, and inclusion level were the major variables affecting cow performance, whereas cow days in milk and experimental duration had no significant impact. Response variables where the covariates explained less than 50 % of the heterogeneity (adjusted $R^{2}$ ) were not considered in the subgroup analysis; unidentified factors not captured in our meta-analysis might have influenced the response of cows to microalgal supplementation.

**Table 3:** Meta-regression of the covariate effect on raw mean differences (RMDs) between microalgae and control treatments.

Dependent variable ( Y , RMD)	Meta-regression parameters ( P value)					Adj. $R^{2}$ (%)
	Intercept	Animal breed	Experimental duration	Microalgae species	Microalgae inclusion level	
					(g kg DM^−1^)	
Intake (kg DM d^−1^)						
Dry matter	- 0.65 (0.04)	- 0.23 (0.45)	- 1.80 (0.29)	0.40 (0.64)	- 0.31 (0.40)	99.60
Crude protein	0.25 ( <0.001 )	0.11 (0.37)	- 0.07 (0.40)	- 0.00 ( <0.001 )	- 0.03 (0.001)	92.70
NDF	- 0.41 (0.07)	- 0.22 (0.49)	0.01 (0.62)	- 0.00 (0.40)	- 0.10 (0.63)	61.23
Rumen fermentation parameters						
Rumen pH	0.06 (0.11)	-	0.06 (0.35)	- 0.18 (0.01)	0.06 (0.24)	97.44
Blood parameters						
Glucose (mg dL^−1^)	4.80 (0.33)	0.23 (0.16)	- 0.83 (0.65)	- 1.20 (0.99)	- 0.57 (0.24)	54.50
Yield (kg d^−1^)						
Milk	4.36 (0.02)	0.07 (0.96)	- 0.30 (0.73)	1.40 (0.72)	1.67 (0.25)	78.52
Protein	0.11 (0.03)	- 0.01 (0.19)	- 0.05 (0.09)	0.00 (0.29)	0.01 (0.26)	71.35
Fat	0.15 (0.01)	- 0.01 (0.11)	- 0.14 (0.11)	0.00 (0.13)	- 0.08 (0.67)	79.45
Composition (%)						
Protein	- 0.21 (0.18)	- 0.04 (0.64)	0.06 (0.14)	0.06 (0.99)	- 0.02 (0.57)	42.36
Fatty acid profile (g 100 g FA^−1^)						
Stearic (C18:0)	- 1.00 (0.09)	3.08 (0.05)	- 2.40 (0.49)	0.50 (0.99)	0.02 (0.15)	76.13
Oleic (C18:1 n-9)	- 8.25 (0.02)	- 0.07 (0.11)	- 3.08 (0.45)	0.70 (0.54)	- 0.63 (0.20)	91.85
Linoleic (LA; C18:2 n-6)	- 0.54 (0.001)	- 0.34 ( - 0.09)	0.04 (0.91)	- 0.07 ( <0.001 )	- 0.01 (0.001)	98.61
Conjugated linoleic (CLA; C18:2 c9t11)	0.17 (0.49)	- 0.27 (0.04)	0.36 (0.52)	0.07 (0.05)	0.19 (0.43)	9.59
α -linolenic (ALA; C18:3 n-3)	- 0.07 (0.02)	- 0.24 ( <0.001 )	0.003 (0.67)	- 0.04 (0.02)	- 0.03 (0.57)	90.14
Eicosapentaenoic (EPA; C20:5 n-3)	0.29 ( <0.001 )	- 0.08 (0.22)	0.06 (0.71)	- 0.01 (0.99)	- 0.02 ( <0.001 )	99.41
Docosahexaenoic (DHA; C22:6 n-3)	1.30 (0.05)	0.45 (0.39)	0.37 (0.49)	- 0.00 (0.10)	0.23 (0.01)	82.58
Total SFA	- 2.09 (0.53)	5.25 (0.03)	- 3.70 (0.47)	- 0.40 (0.49)	- 0.65 (0.17)	2.80
Total PUFA	0.34 (0.30)	- 0.53 (0.36)	0.93 (0.57)	0.05 (0.97)	0.25 (0.13)	40.54
n-6	- 0.03 (0.002)	- 1.38 (0.002)	0.10 (0.96)	- 0.03 ( <0.001 )	- 0.25 (0.26)	98.16
n-6 : n-3	- 0.31 (0.18)	- 0.27 (0.85)	- 0.21 (0.21)	0.06 (0.07)	- 0.39 (0.33)	72.77

RMD: raw mean difference, CI: confidence interval of RMD, P : probability, IU: international unit, MUFA: monounsaturated fatty acid, PUFA: polyunsaturated fatty acid, SFA: saturated fatty acid; n-3: omega-3 FA, n-6: omega-6 FA.

### 3.4 Subgroup analysis

**Figure 5:**
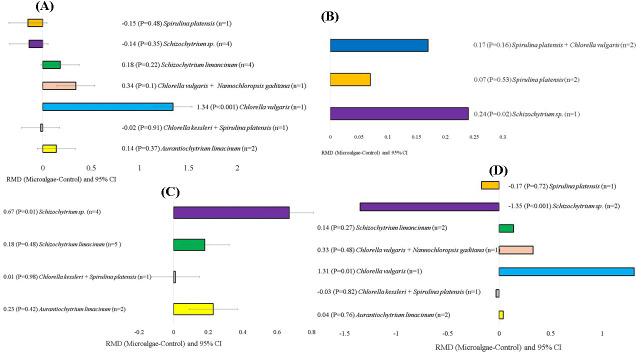
Subgroup analysis (subgroup = microalgae species) of microalgae effects on the diet of small ruminants. RMD: raw mean difference between microalgae treatment and control. **(A)** C18:2 n-6 (g 100 g FA^−1^), **(B)** rumen pH, **(C)** C22:6 n-3 (g 100 g FA^−1^), **(D)** n-6 (g 100 g FA^−1^).

**Figure 6:**
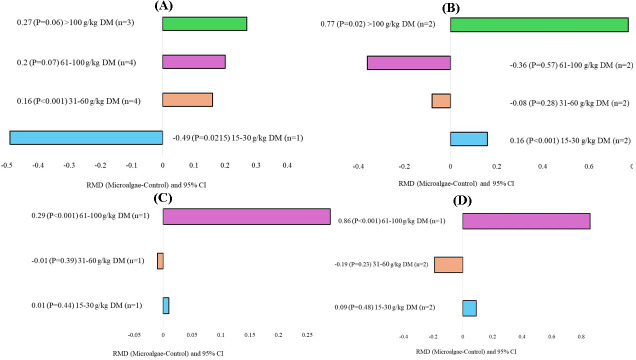
Subgroup analysis (subgroup = inclusion level) of microalgae effects on the diet of small ruminants. RMD: raw mean difference between microalgae treatment and control. **(A)** Crude protein intake (g d^−1^), **(B)** C18:2 n-6 (g 100 g FA^−1^), **(C)** C20:5 n-3 (g 100 g FA^−1^), and **(D)** C22:6 n-3 (g 100 g FA^−1^).

**Figure 7:**
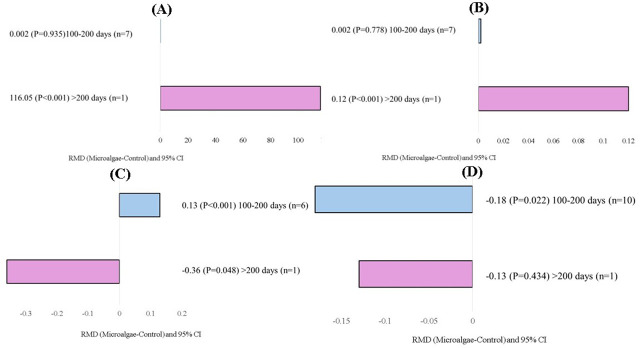
Subgroup analysis (subgroup = experimental duration) of microalgae effects on the diet of small ruminants. RMD: raw mean difference between microalgae treatment and control. **(A)** C15:0 (g 100 g FA^−1^), **(B)** C17:0 (g 100 g FA^−1^), **(C)** C16:1 (g 100 g FA^−1^), **(D)** n-6 (g 100 g FA^−1^).

**Figure 8:**
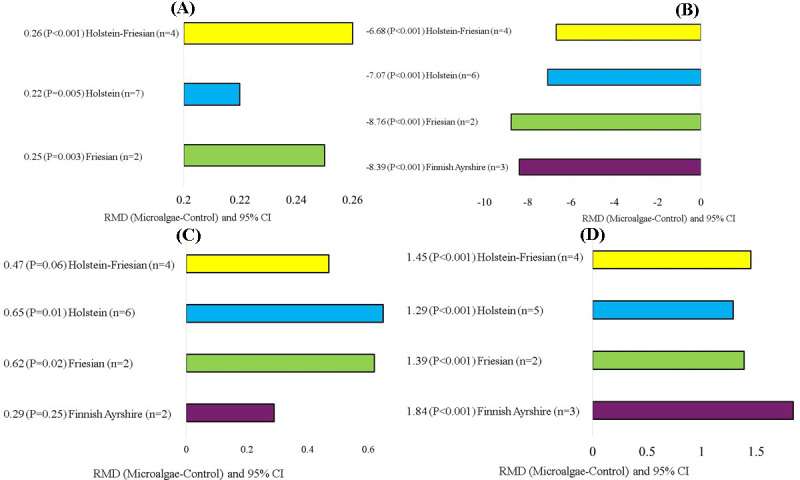
Subgroup analysis (cow breed) of the effect of microalgae on the diet of small ruminants. RMD: raw mean difference between microalgae treatment and control. **(A)** C18:2 n-6 (g 100 g FA^−1^), **(B)** saturated fatty acid (SFA; g 100 g FA^−1^), **(C)** C18:2 c9t11 (g 100 g FA^−1^), **(D)** n-6 (g 100 g FA^−1^).

The microalgae species, level of incorporation, experimental duration, and animal breeds that affected milk production and quality are shown in Figs. 5, 6, 7, and 8, respectively. When evaluating the covariate microalgae species, incorporation of *Schizochytrium* sp. into the diet of dairy cows increased DHA (RMD = 0.67 g 100 g FA^−1^; P=0.01 ) and decreased n-6 content (RMD = - 1.35 g 100 g DM^−1^; P<0.001 ), whereas *Chlorella vulgaris* increased n-6 (RMD = 1.31 g 100 g FA^−1^; P=0.01 ) and C18:2 n-6 (RMD = 1.31 g 100 g DM^−1^, P<0.001 ) levels. For the level of incorporation covariate, the lower level of microalgae inclusion (15–30 g kg DM^−1^) increased C18:2 n-6 (RMD = 0.16 g 100 g FA^−1^; P<0.001 ). The medium incorporation of microalgae (31–60 g kg DM^−1^) increased CPI (RMD = 0.16 kg d^−1^; P<0.001 ). Higher incorporation of microalgae (61–100 g kg DM^−1^) increased the C20:5 n-3 (RMD = 0.29 g 100 g FA^−1^; P<0.001 ) and C22:6 n-3 (RMD = 0.86 g 100 g FA^−1^, P<0.001 ) contents. Excessive incorporation of microalgae ( >100 g kg DM^−1^) into the diet of cows increased only C18:2 n-6 (RMD = 0.77 g 100 g FA^−1^, P=0.02 ). Regarding the effects of the animal breed covariate, microalgae incorporation into Holstein and Friesian cows significantly increased C18:2 n-6 (RMD = 0.26 g 100 g FA^−1^; P<0.001 ), C18:2 c9t11 (RMD = 0.65 g 100 g FA^−1^, P=0.01 ), and n-6 (RMD = 1.29 g 100 g FA^−1^, P<0.001 ), and significantly decreased saturated FA (SFA; RMD = - 7.07 g 100 g FA^−1^, P<0.001 ). For the experimental duration covariate, longer supplementation with microalgae ( >200 d) increased C15:0 and C17:0 but decreased C16:1. The duration of medium supplementation increased C16:1 and decreased n-6.

## 4 Discussion

### 4.1 Description of studies considered in the analysis

Beyond their ecological importance, microalgae have gained interest as low-cost animal feed supplements (Altomonte et al., 2018). In the present meta-analysis, almost 90 % of the included studies were conducted between 2015 and 2020. This could be due to the emerging focus on addressing environmental challenges and promoting sustainable agricultural practices. The studies were conducted in a few countries but encompassed cows from three continents (Europe, the Americas, and Asia). Africa was not included in the present meta-analysis; however, studies on microalgae incorporation in other ruminant species (goats) have been reported (Kholif et al., 2020). These differences could be due to climatic differences and the strong dairy industry in Europe, which focuses on product quality (Britt et al., 2018).

### 4.2 Overall effects of microalgae on lactating performances of cows

Dry matter intake is a critical factor influencing dairy cow productivity (Manzocchi et al., 2020). The decreased microalgae intake by cows could be attributed to their low palatability, which is due to their taste and odor and their physically dry and finely powdered structure (Lamminen et al., 2017). The palatability of microalgae can be enhanced by pelleting the dairy cow concentrate diets (Lamminen et al., 2017).

Furthermore, considering the usual level of microalgae in diets, an impact on fiber (NDF) digestibility was not expected, except in diets with a high concentrate. Enhanced total-tract NDF digestibility results from a slower passage rate and longer retention time of digesta owing to decreased feed intake. Additionally, reduced intake may stem from adverse effects on ruminal fiber digestibility, leading to increased satiety. Alterations in the molar VFA proportion, particularly in the acetate:propionate ratio, are the outcome of long-chain PUFA n-3 supplementation (Shingfield et al., 2012) and can be attributed to changes in the rumen bacterial community. Research suggests that fiber-degrading microorganisms negatively affected by PUFA supplementation can be replaced by strains that are less susceptible to PUFA's toxic effects, demonstrating the rumen microbiota's capacity to adapt to environmental changes, seemingly without compromising ruminal NDF digestibility (Dewanckele et al., 2020), at least at low levels of PUFA inclusion. Similar results were reported by Marques et al. (2019). Moreover, the improved production of isovalerate can be attributed to the microbial fermentation of branched-chain amino acids (valine and leucine) present in some microalgal species.

The incorporation of microalgae into cow diets aims to improve milk yield and quality. In the present meta-analysis, milk yield was not affected despite the decreased intake of DM and OM, which could be due to the nutrient density of microalgae or metabolic adjustments to maintain milk production when cows face variations in their dietary conditions. The decrease in milk fat in cows supplemented with microalgae could be due to biohydrogenation intermediates, such as t10c12 CLA, which can inhibit milk fat synthesis (Dewanckele et al., 2020). However, this FA was not evaluated in the present meta-analysis.

Stearic acid (C18:0) represents the end product of biohydrogenation in the rumen, whereas trans-vaccenic acid (C18:1 t11) is the final product when ruminal biohydrogenation is reduced (Boukrouh et al., 2023b, 2024c; Boukrouh, 2023; Dewanckele et al., 2020). The microalgae in the present meta-analysis blocked biohydrogenation in the first step and led to increased C18:1 t11 and C18:2 c9t11 CLA as biohydrogenation intermediates.

### 4.3 Microalgae species

Microalgae are a rich source of essential long-chain PUFA, including C22:6 n-3 (DHA) (Chen et al., 2023). The reason for the higher DHA content in the milk of cows supplemented with microalgae, especially cows supplemented with *Schizochytrium* sp., is probably its bypass to milk due to the protection from biohydrogenation (Boukrouh et al., 2023a). The reason for the increased linoleic acid C18:2 n-6 when *Chlorella vulgaris* was supplemented to cows could be due to the higher concentration of *Chlorella vulgaris* in this FA and the inhibition of their ruminal biohydrogenation (Petkov and Garcia, 2007).

### 4.4 Level of incorporation

Meta-regression analysis revealed that the level of inclusion was a limiting factor influencing the variability in the study results regarding the impact of microalgae supplementation on dairy cow performance. Subgroup analysis highlighted that incorporation of 60–100 g kg DM^−1^ of microalgae is needed to improve cow milk nutritive value concerning its content of fat, linoleic C18:2 n-6 (LA), linolenic C18:3 n-3 (LNA), C20:5 n-3 (EPA), and C22:6 n-3 (DHA) acids. LA and LNA are the precursors to conjugated linoleic acids and long-chain FA (EPA and DHA), which are beneficial to health (Acosta Balcazar et al., 2022). Excessive incorporation of more than 100 g kg DM^−1^ is unnecessary, as despite the increased CP intake, milk protein yield did not increase, probably because of a dilution effect. Moreover, decreasing the amount of microalgae supplementation may have environmental implications, such as impacts on efficient energy and water use. Additionally, optimization of the level of microalgae incorporation and cost-effectiveness should be considered to avoid the fact that supplements may be more expensive than traditional feed ingredients (Rafa et al., 2021). From this perspective, recommendations encourage additional studies with similar experimental designs to investigate the impact of microalgae supplementation on the economic aspects of cow milk production, especially because microalgae cultivation is in the development phase and faces several challenges linked to environmental conditions (temperature, light intensity, and pH), energy consumption, and water use sustainability (Peter et al., 2022).

### 4.5 Experimental duration

The experimental duration was expected to be an important factor affecting the response of cows to microalgae incorporation. It was expected that there would be an adjustment period during which cows might gradually experience the physiological and nutritional advantages of microalgae supplementation, with these benefits potentially increasing with prolonged supplementation (Manzocchi et al., 2020). In a shorter period of supplementation, immediate changes may not be noticeable, and it may take time for the cow's metabolism to adjust to dietary microalgae supplementation. This explains the absence of a shorter supplementation duration ( <100 d) for milk production and quality. Extended periods of supplementation ( >200 d) could provide time for adaptation in both the rumen microbial population and the metabolic pathways, potentially resulting in pronounced variations in milk composition. However, in the present meta-analysis, a longer experimental duration did not affect milk or FA profiles. These results suggest that supplementing cows with microalgae for an extended period had no further effect on substantial dairy cow performance, indicating that a short period of less than 100 d could be used as a supplementation period, taking into consideration the higher microalgae price, especially at the inclusion level of 61–100 g kg DM^−1^, which is mandatory to obtain an improvement in the FA profile. As microalgae are a potential source of n-3 FA, a decrease in n-6 and n-6 : n-3 was expected and testified to the importance of microalgae in improving consumer health (Chen and Liu, 2020).

### 4.6 Animal breed

The variations in milk yield among different bovine breeds across diverse geographical regions may be attributed to a combination of factors, including genetic and environmental influences, as well as management practices (Ammer and Gauly, 2020). The effect of cow breed on their response to microalgae incorporation may exist due to differences in the rumen microbial community (Bainbridge et al., 2016). The reason for the reduced C14:1 and C16:1 levels in Chinese–Holstein cattle could be the possible differences in mammary desaturase activity between breeds (Carroll et al., 2006; Li et al., 2016). The index D914 has been suggested to be the best indicator of desaturase activity because the product (C14:0) originates almost exclusively from *de novo* synthesis in the mammary gland (Lock and Garnsworthy, 2003). Stearic acid (C18:0) represents the end product of the ruminal biohydrogenation process, whereas vaccenic acid C18:1 t11 is the final product in the case of inhibition of ruminal biohydrogenation (Dewanckele et al., 2020). The reason for increased C18:0 and SFA for Chinese–Holstein cows and reduced SFA with a parallel increase in vaccenic acid for the Friesian breed could be due to the differences in ruminal microbial communities, which affected ruminal biohydrogenation differently between the two breeds in response to dietary fat present in microalgae.

### 4.7 Investigation of heterogeneity and publication bias

Considerable heterogeneity was found in this meta-analysis, and the meta-regression analysis supported the presence of other sources of variation apart from those engendered by the considered covariates. Publication bias is generally caused by the tendency of reviewers and journal editors to favor the publication of studies showing significantly positive results rather than those reporting negative findings (Nosek et al., 2022). It is widely recognized that publication bias tends to impact the configuration of the funnel plot, often excluding small studies with non-significant results. As a result, as shown in the funnel plots of the trim-and-fill analysis for the two variables (C14:1 and CPD), the lower-left parts of the funnel plots are less populated and are the ones that are filled with excluded studies (Field and Gillett, 2010). For the other variables, Rosenthal's FSN were higher than the threshold required to confirm the pooled estimate results. Therefore, there was no evidence of publication bias in this meta-analysis. The substantial difference between dairy cows in the experimental and control groups remained statistically significant, and any conceivable bias would necessitate a considerable number of unpublished, non-significant studies to alter this result.

It is important to highlight that some microalgae species or inclusion levels have been examined in only a limited number of studies, often just a single one. This finding emphasizes the need for extensive research to fully explore these relationships. Future studies should aim to fill these knowledge gaps by encompassing a wider variety of goat breeds, experimental settings, and microalgal species. Such comprehensive research would enhance the existing evidence base and offer more definitive insights into how microalgae supplementation impacts dairy cow performance.

## 5 Conclusion

The exploration of microalgae as feed supplements represents a promising perspective in contemporary research to improve livestock production and quality. Although the findings indicated some heterogeneity, publication bias was reported for only one variable. The results of the present meta-analysis emphasize the potential use of microalgae to improve the nutritional profile of milk by improving health-beneficial fatty acids. Meta-regression analysis demonstrated that certain covariates accounted for variations in the results. An optimal fatty acid profile was achieved with *Schizochytrium* sp. when microalgae were integrated into the diets of Holstein and Friesian cows. The incorporation of 61–100 g kg DM^−1^ improved the fatty acid profile of milk. Milk yield was not affected despite lower microalgae intake. These results provide valuable insights for animal nutritionists and policymakers, guiding evidence-based decisions on the effective utilization of microalgae feed supplements to enhance cow performance.

## Supplement

10.5194/aab-69-101-2026-supplement The supplement related to this article is available online at https://doi.org/10.5194/aab-69-101-2026-supplement.

## Data Availability

The datasets and software codes presented in this study are available upon request from the corresponding author.
