# Supplementary material for: A systematic review and meta-analysis of the effects of inclusion of microalgae in dairy cows' diets on nutrient digestibility, fermentation parameters, blood metabolites, milk production, and fatty acid profiles
Source: Arch Anim Breed. 2026 Feb 10;69(1):101–15. doi: 10.5194/aab-69-101-2026 (PMC13105246; doi:10.5194/aab-69-101-2026)
Supplement: The supplement related to this article is available online at https://doi.org/10.5194/aab-69-101-2026-supplement. [file aab-69-101-2026-supplement.pdf]

Supplement of Arch. Anim. Breed., 69, 101–115, 2026
https://doi.org/10.5194/aab-69-101-2026-supplement
© Author(s) 2026. CC BY 4.0 License.

Open Access
Archives Animal Breeding

Supplement of

A systematic review and meta-analysis of the effects of inclusion of microalgae in dairy cows' diets on nutrient digestibility, fermentation parameters, blood metabolites, milk production, and fatty acid profiles

Soumaya Boukrouh et al.

Correspondence to: Soumaya Boukrouh (soumaya.boukrouh@um6p.ma, soumaya.boukrouh@gmail.com)

The copyright of individual parts of the supplement might differ from the article licence.

Supplementary Table S1. Summary of the studies included in the meta-analysis.

Reference	Country	Number of animals	Animal breed	Days in milk (days)	Experimental Duration (days)	Microalgae specie	Inclusion level (g/kg DM)
Boeckaert et al., 2008	Belgium	4	Holstein	<90	<100	*Schizochytrium sp.*	>100
Da Silva et al., 2016	Brazil	20	Holstein	<90	<100	*Prototheca moriformis*	61-100
Huntington et al., 2020	UK	20	Holstein	<90	<100	*Schizochytrium limancinum*	<15
Fougère et al., 2018	France	12	Holstein	<90	<100	*Schizochytrium sp.*	61-100
Franklin et al., 1999	USA	30	NR	>90	100-200	*Schizochytrium sp.*	31-60
Liu et al., 2020	China	36	Chinese-Holstein	NR	NR	*Schizochytrium sp.*	<15
Manzocchi et al., 2020	Switzerland	12	NR	>90	>200	*Arthrospira platensis*	31-60
Marques et al., 2019	Brazil	24	Holstein	>90	100-200	NR	<15
Moran et al., 2017	Italy	36	Friesian	>90	100-200	*Aurantiochytrium limacinum*	<15
Moran et al., 2018	Italy	24	Friesian	>90	100-200	*Aurantiochytrium limacinum*	<15
Lamminen et al., 2017	Finland	6 and 8	Finnish Ayrshire	>90	100-200 and >200	*Spirulina platensis alone, and added to Chlorella vulgaris*	31-60
Lamminen et al., 2019	Finland	8	Finnish Ayrshire	>90	100-200	*Spirulina platensis*	31-60
Lamminen et al., 2019	Finland	4	Finnish Ayrshire	>90	100-200	*Spirulina platensis, Chlorella vulgaris and their mix*	61-100 and >100
Póti et al., 2015	Hungary	16	Holstein-Friesian	>90	100-200	*Chlorella kessleri + Spirulina platensis*	<15

Till et al., 2019	UK	60	Holstein-Friesian	<90	<100	*Schizochytrium limancinum*	<15
Till et al., 2020	France	32	Holstein	>90	100-200	NR	15-30
Vanbergue et al., 2018	France	30	Holstein	>90	<100	NR	15-30

NR not reported.
